# The transcatheter aortic valve implementation (TAVI)—a qualitative approach to the implementation and diffusion of a minimally invasive surgical procedure

**DOI:** 10.1186/s13012-015-0330-1

**Published:** 2015-10-06

**Authors:** Sebastian Merkel, Michaela Eikermann, Edmund A. Neugebauer, Stephan von Bandemer

**Affiliations:** Institute for Work and Technology (IAT), Munscheidstr. 14, 45886 Gelsenkirchen, Germany; Institute for Research in Operative Medicine (IFOM), Ostmerheimer Str. 200 Haus 38, 51109 Köln, Germany

**Keywords:** Implementation, Diffusion, Hospital, Cardiology, Cardiothoracic surgery, Barriers and facilitators

## Abstract

**Background:**

The transcatheter aortic valve implantation (TAVI), a minimally invasive surgical procedure to treat patients with severe symptomatic aortic stenosis, showed a rapid diffusion in Germany compared to the international level. The aim of this study is to identify and analyze factors affecting the implementation and diffusion of the procedure in hospitals using a qualitative application of the diffusion of innovations theory.

**Methods:**

We conducted problem-centered interviews with cardiologists and cardiac surgeons working in German hospitals. The multi-level model “diffusion of innovations in health services organizations” developed by Greenhalgh et al. was used to guide the research. Data was analyzed using content and a thematic analysis.

**Results:**

Among the ten participants who were interviewed, we found both barriers and facilitators related to the innovation itself, system readiness and antecedents, communication and influence, and the outer context. Key issues were the collaboration between cardiologists and cardiac surgeons, reimbursement policies, requirements needed to conduct the procedure, and medical advantages of the method.

**Conclusions:**

The findings show that there are multiple factors influencing the diffusion of TAVI that go beyond the reimbursement and cost issues. The diffusion of innovations model proved to be helpful in understanding the different aspects of the uptake of the procedure. A central theme that affected the implementation of TAVI was the collaboration and competition between involved medical departments: cardiology and cardiac surgery. Against this background, it seems especially important to moderate and coordinate the cooperation of the different medical disciplines.

## Background

If medical innovations show a positive effect on the quality of care and/or the treatment cost, they can lead to an increased productivity of hospitals and healthcare systems. However, if innovations in healthcare show positive outcomes, they seem to spread relatively slowly [1]. The reasons for a fast or slow diffusion are both complex and multilayered. It could be assumed, for example, that devices or drugs with strong clinical evidence spread faster than those with lagging evidence. However, several studies have proven that that is not always the case [2–4].

The transcatheter aortic valve implantation (TAVI)^1^ is a relatively new method to treat patients with severe symptomatic aortic stenosis (AS). TAVI represents a minimally invasive alternative in comparison to the current standard AS treatment, which is surgical aortic valve replacement (AVR). With TAVI, a replacement valve is introduced through an artery via a small incision, thus requiring no surgery. Supported by results of randomized trials, TAVI can be seen as “the new standard of care for patients with symptomatic AS who are deemed ‘inoperable’” [5, 6]. The first-in-man implantation was performed in 2002, and by 2007, two devices were certified in Europe [7]. In 2010, the method was completely reimbursed by the German therapy-specific diagnosis-related group (DRG). In the same year, the first results of a randomized controlled trial comparing TAVI versus medical therapy versus AVR were released [5]. Since 2007, the procedure has shown significant adoption rates in Germany compared to the international level. In Europe, almost 45.9 % of all TAVI have been implanted in German hospitals, thus making it the largest market in the EU [8, 9]. Starting with a total of 68 implanted devices, this amount had increased up to 10,814 by 2013. The same is true considering centers performing the method: With 22 centers in 2008, and 93 in 2013, Germany had the most centers in Europe (see Table 1).

**Table 1:** Diffusion of TAVI in Germany from 2006 to 2013 [30–33]

	2006	2007	2008	2009	2010	2011	2012	2013
Total amount of TAVI	68	239	1,463	3,411	5,799	7,681	9,271	10,814
Amount of centers	2	10	22	61	80	90	91	93
Centers performing TAVI without an on-site cardiac surgery	-	-	-	-	14	18	18	17

This development has been repeatedly criticized [8, 10]. Critics argue that the method had begun to diffuse widely, while at the same time, valid clinical evidence was still missing, especially in the consideration of which patients should be receiving the intervention [8]. With increasing uptake, TAVI was also performed by hospitals which did not meet the recommendations of the national and European cardiac and cardiothoracic societies and associations regarding requirements necessary for conducting the method [9–11]. This includes the involvement of a “Heart-Team” involving cardiologists, cardiac surgeons, and anesthetists and having an on-site department of cardiac surgery. Storz-Pfennig et al. summarize the development: “The criticisms support the view that the technology was, and still is being, used beyond proper indications and in treatment facilities not adequately equipped for emergencies.” [9]. A new guideline passed by the German Federal Joint Committee in January 2015 has set limits for hospitals performing TAVI: Only those that have a department of cardiology and cardiac surgery shall be allowed to provide the intervention [12].

Improving the diffusion of cost-effective technologies, while at the same time preventing the uptake of medical technologies that are of “doubtful value” for healthcare systems, is the purpose of health technology assessment (HTA) [13]. HTA is the systematic evaluation of effects and impacts of health technology from a multidisciplinary perspective covering medical, social, legal, ethical, and economic aspects. The approach has originally emerged as a response by policy makers to the uncontrolled diffusion of medical devices and products [14]. Diffusion research, including drivers and barriers affecting the uptake of health technologies, is an important aspect of HTA. In turn, the healthcare sector has played a dominant part in the history of implementation and diffusion research. The pioneer work of Coleman, Katz, and Menzel, who studied the spread of a new drug among physicians, marks the first of numerous studies analyzing the factors and determinants influencing the adoption, implementation, and the spread of innovations in the healthcare sector [15]. Recent studies describe four core domains playing a role within the implementation and diffusion process: the structural, organizational, individual, and innovation level [16, 17]. Each level encompasses multiple determinants affecting the implementation success and the diffusion rate. For example, Fleuren et al. found 50 potentially relevant determinants of innovation processes [17]. Following Blume’s argumentation that every medical innovation has its own “career,” these determinants differ with respect to the research subject [18].

Several theories and models can be applied to better understand the implementation and diffusion of innovations in healthcare—most notably, Everett M. Rogers’ Diffusion of Innovations (DOI) theory [19], as well as the diffusion of innovations model described by Greenhalgh et al. [20]. Both have been adopted in numerous studies analyzing the implementation and diffusion of innovations in healthcare (see, for example, [1, 21, 22]). Roger’s DOI theory has been picked up by multiple researchers in various contexts and disciplines to explain the spread of emerging innovations. In short, the approach explains why certain innovations spread more easily than others. For instance, the individual’s perceived attributes of an innovation (relative advantage, complexity, compatibility, observability, and trialability) account for 49 to 87 % of the adoption variation [19]. The nine components of the multi-level model detailed below, described by Greenhalgh et al., focus mainly on the healthcare sector. (1) The innovation itself: As shown by Rogers, the innovation’s attributes can explain much of the variance in the adoption rate of innovations. In addition to the five core attributes described in the DOI theory, Greenhalgh et al. add the potential of reinvention, risk, task issues, ease of use, knowledge required to use it, and support [20]. (2) The potential adopter: Adoption is considered as an active process; one’s past experiences affect the adoption decision, and persons are more or less risk-averse. (3) Communication and influence: The adoption decision can be actively influenced by opinion leaders or champions, or it can occur by copying others. (4) System antecedents of innovation include structural factors (e.g., the size of an organization), the capacity for new knowledge (e.g., pre-existing knowledge or skills), and the context for change (e.g., risk-taking climate). (5) System readiness for innovation covers the “organization’s ability to adopt a particular innovation” [23]. (6) The outer context: Both the economic and social situation, as well as competing companies and institutions, can have an effect on adoption decision. (7) The process of assimilation: Innovations “have a ‘hard core’ (the irreducible elements of the innovation itself) and a ‘soft periphery’ (the organizational structures and systems required for the full implementation of the innovation)” [20]; adopting the soft elements can result in a complex and non-linear process. (8) The implementation process: A successful implementation can be affected by dedicated resources or external collaboration. (9) The linkage between the components.

One explanation for the rapid diffusion of TAVI is found in the reimbursement policies and, accordingly, the cost of the procedure [8–10]. It can be assumed, however, that this is only one factor among many others. The main purpose of our study is to identify the factors affecting the uptake of TAVI. The case of TAVI can provide deeper insights into the adoption, implementation, and diffusion of medical innovations in hospitals in general.

## Method

### Data collection

We used the model provided by Greenhalgh et al. to guide our research and to analyze the data. Based on these theoretical implications, along with the fact that qualitative approaches have continuously gained relevance when studying complex interventions [24], we used problem-centered interviews to gather our data [25]. The problem-centered interview represents a discursive procedure “to gather objective evidence on human behavior as well as on subjective perceptions and ways of processing social reality” [25]. The method was developed by Witzel, who summarizes it as “a theory-generating method that tries to neutralize the alleged contradiction between being directed by theory or being open-minded so that the interplay of inductive and deductive thinking contributes to increasing the user’s knowledge.” [25]. Though we do not know any comparable work using this instrument to study the adoption, implementation, and diffusion of innovations in healthcare, the method has been used in market research to gain a better understanding of factors influencing the adoption of innovative products. The interviews were structured as follows: A pre-formulated introductory question or statement (“Please tell us about the implementation process of TAVI within your hospital; from the very beginning until today.”) stimulates the interviewees to talk about the implementation process within the organization. While the interviewer only plays a passive role during the first phase of the interview, the second phase consists of general exploration and ad hoc questions. The interviewer “can ask questions which allow the thread of the story to be further spun” [25]. To compare the interviews with each other, we used an interview guide based on the theoretical framework and on additional material about the research subject. The interview guide was piloted in two interviews prior to data collection. All interviews were conducted in German, and all quotes cited within this paper were translated.

### Sample size and recruitment

While there is no general agreement regarding the sample size for the problem-centered interview, between 7 to 30 interviews is suggested based on previous research [26]. We did not define a specific sample size a priori; the sampling of new participants continued until we achieved redundancy. We interviewed cardiologists and cardiac surgeons working in hospitals who also have experience in using TAVI for at least 1 year. To identify potential participants, a list of hospitals using the procedure was drawn from the hospital’s quality reports^2^. The respondents were chosen purposefully and randomly. Because it proved difficult to include participants, we did not aim for maximum variation. The purposeful sampling only covered the size of the hospital, using the amounts of beds as a proxy, and the hospital type (whether it was a university hospital or not). The potential participants were contacted by email, which contained an informational document explaining the aim and the method of the study and that the identification of participating persons will not be possible after the analyses. Depending on the time schedule of the interviewees, either a face-to-face or a telephone interview was set up. The interviews were carried out by a trained researcher between February and March 2014, digitally recorded and transcribed verbatim.

### Analysis of the data

Each transcript was analyzed using qualitative content analysis, which was then organized in four steps: (1) There was a familiarizations process with the data to gain a deeper understanding. This included reading through the transcript multiple times. (2) Using an inductive and deductive approach, categories were generated that helped to compromise the data without losing information. Based on the model by Greenhalgh et al. [20], we set up an initial coding scheme that used all of the above mentioned nine components as main categories for the coding process. (3) We used an inductive approach to developed sub-categories based on the data and to ensure that all aspects were covered. After coding approximately 40 % of the data, we revised and refined the coding scheme. The final coding scheme did not cover all main categories. We focused on the innovation itself, communication and influence, system readiness, system antecedent, and the outer context. (4) Lastly, the interviews were analyzed using MAXQDA11. The development of the coding scheme and the analysis of the gathered data were undertaken by the researcher who conducted the interviews.

### Anonymization

Though anonymization does not receive much attention in qualitative studies [21], it has to be seen as a central aspect and leads to multiple challenges. On the one hand, an insufficient anonymization can cause harm to the participants and the organizations they work for. On the other hand, too much anonymization affects the results of the research process [21]. We decided to remove all evidence that could lead to an identification of either the interviewee or the hospital he or she works for. Since only around 4 % of all hospitals in Germany work with TAVI, we deleted whole passages and used synonyms. The deletion includes such information as hospital size, type of hospital, the date the procedure had been implemented, the sex of the participant, and the hierarchical position, as well as any references that would indicate the location of the clinic.

## Results

In total, we conducted ten interviews across nine sites, three of which were university hospitals. Eight interviews were face-to-face and two via telephone. The interviews lasted between 20 to 35 minutes, the average time length being approximately 25 min. The sample is characterized by an over representation of cardiologists (nine out of ten respondents). The inductive approach revealed several factors that were mentioned by the interviewees within the following themes: collaboration between cardiologists and cardiac surgeons, reimbursement policies, requirements and skills needed to conduct the procedure, and medical advantages of the method. All of these themes are reflected by the model. We could identify potential facilitators and barriers to the adoption, implementation and diffusion related to the innovation itself, communication and influence, system readiness for innovation and system antecedents for innovation, the implementation process, and the outer context.

### Innovation level

After the initial knowledge phase and getting in touch with the procedure for the first time, most participants rejected TAVI: “What nonsense. It cannot work this way.” (Cardiologist 4). After reconsideration and further discussion of TAVI, e.g., at internal meetings, the decision for implementation was made.

The respondents described multiple advantages of TAVI compared to the standard surgical procedure and drug treatment. Our interviewees generally mentioned medical outcomes and the improved quality of life of patients who underwent TAVI as an advantage. Several participants gave examples of how fast the patients recovered after the intervention and that the patients were able to leave the hospital much earlier compared to the standard surgery. In terms of medical outcomes, the results were not only perceived as better compared to the “classical” surgical procedure but were also directly visible to the physician and to the patient, as well as his or her relatives.

“You must have seen this, indeed. Patients are responsive right after the procedure, have little pain, at worst a bit of a pain in the groin, and can be mobilized in the evening or the next day… But they are responsive directly after the procedure, can eat, drink, talk with their families. They can be mobilized on the first day, third day down from the ICU, out of the house on the fifth day. You won’t find this with any other surgical procedure.” (Cardiologist 3)

“But it is an intervention in which the patient is basically walking around the next day. And with heart surgery that is, of course, very, very different. That’s it, especially for older persons… And I think the 80-year-old patient must quickly get out of the hospital and back into his environment and considering this, such a method has, of course, a huge advantage.” (Cardiologist 1)

One participant perceived the procedure as “highly complex” (cardiologist 7), a view that was shared. Even though TAVI proved compatible with existing practices, the method was seen as difficult to perform. Responses commonly indicated that TAVI required new knowledge and intensive training.

“But at the time we introduced that, when the treatment was new, each component had to be learned first […]” (Cardiologist 5)

“This is certainly a different dimension compared to a normal coronary intervention because you work with much, much thicker systems in marginal thin vessels.” (Cardiologist 5)

The respondents felt that TAVI has a steep learning curve. As the participant below estimated, it takes around 50 to 60 interventions to master the procedure; a number that was verified by another interviewee.

“I would say, until someone can reasonably do it, minimum numbers are not defined, but I think you need about 50 to 60 procedures…” (Cardiologist 4)

The risk of the procedure was linked to the available evidence. The respondents perceived the evidence base as good concerning patients aged 75 and older but found it not sufficient regarding younger patients. They arrived at the conclusion that the standard surgical procedure was still seen as superior with respect to this patient group. The criticism was driven by the lack of empirical knowledge concerning the duration of the implanted aortic valves.

### Communication and influence

The physicians referred to two main sources of information. They consulted professional journals and national and international congresses, as well as opinion leaders at both the national and international level or “frontiers” as one interviewee said. Some of the respondents actively established contact with either one of the opinion leaders or with a medical device company in order to learn about the procedure. In the case of the medical device company that developed the artificial valves, they offered information and training sessions for the staff involved and promoted the implementation of the method. In one case, an interviewee referred to a conflict between two of the involved opinion leaders, where the cardiac surgeon rejected the method and the cardiologist supported it. The head physicians of the department of cardiology and cardiac surgery were reported to have played key roles in the implementation within the hospital, whereas support by the administration was only reported in one case. While some physicians stated that patients explicitly asked for TAVI instead of the standard treatment, this was perceived as rarely the case. More common seemed to be the fact that relatives and friends heard about the procedure.

“The patients who inform themselves and ask for it are those to whom one would offer it reluctantly because these are the younger patients…” (Cardiologist 4)

Two respondents mentioned that the hospitals they work for organize events to facilitate the dissemination of TAVI, either targeting general practitioners or patients. In contrast, one participant emphasized that there are no efforts in “advertising.”

### System readiness and system antecedents

Pressure for change was widely reported by the respondents. The interviewees named a combination of different aspects as reasons for this pressure, most notably the organizational self-image, as well as competition between hospitals. Hospitals that consider themselves as innovative were reported to having wanted to implement the procedure and be among the early adopters, as the following respondent replied:

“Then there are early adopters, who quickly adopt good and promising practices, that’s us. And then there are the laggards, late adopters, and that is what we do not want to be.” (Cardiologist 3)

The interviewees referred to organizational requirements needed to implement the procedure. In multiple interviews, the heart team was mentioned as a central player in considering the implementation of the method. The views of the participants differ considering the composition of the heart team. While in most interviews it was mentioned that a cardiac surgeon, an interventional cardiologist, and an anesthesiologist should be present during the procedure, the comment below illustrates that reality could look different:

“…the heart team should not exist merely on paper. In other words, that there is a surgeon somewhere who is only on standby, or who says ‘yeah, sure’.”(Cardiologist 8)

The opinions about the required staff changed according to the type of hospital. Interviewees working in university hospitals found it inevitable to have an in-house department for cardiovascular surgery, while those working in smaller and specialized hospitals did not share this view. Hospitals without such a department sought expertise from outside and hired an external surgeon especially for TAVI.

“This cannot be managed by a normal house where only standard operations are conducted and without a cardiac surgery. You have to say this. And houses that do it, which have the logistics, emphasize the safety aspect and that they cover the all safety issues…” (Cardiologist 4)

The most evident factor mentioned by nearly all interviewees was the internal collaboration with other medical departments, most notably between cardiologists and cardiac surgeons. Many respondents described this struggle as a factor influencing the adoption decision and slowing down or interrupting the implementation process, as the statements below indicate:

“These are the nuts and bolts… Because, often the teamwork of cardiologists and cardiac surgeons is not given…This is a major problem because the interventional cardiology has expanded into many areas of heart surgery…” (Cardiologist 5)

“Because, especially in the initial phase… we failed because of the resistance of the cardiac surgery without which, of course, it didn’t work at that time, or still does not work.” (Cardiologist 1)

The respondents commented that TAVI created an issue of competence. While the cardiologists felt eager to implement the method, the cardiac surgeons were described as reluctant and refusing. Some interviewees felt that this was because of a “restructuring” of those two medical disciplines. More and more treatments, which originally belonged to the field of work of the cardiac surgeons, have now become the responsibility of the cardiologists, as the following statement indicates:

“With the introduction, I think there was concern in all houses… I believe this was the case everywhere… It’s psychological. You delve into the innermost of the heart or in the core competence of cardiac surgery. The colleagues got used to the fact that cardiologist do more and more. They have taken pacemakers and defibrillators, all previously done by surgeons… But if someone wants to have a new aortic valve, then he needs to ask us.” (Cardiologist 4)

After the conflict had been solved, one respondent felt that “…the TAVI program has actually resulted in a much closer cooperation with the cardiac surgery.” (Cardiologist 1). The interviewee described that after initial denial, a restructuring took place which created space for new cooperation between both departments. The implementation phase took about 1 year, as some of the participants confirmed.

### The outer context

Concerning the extra-organizational context, the respondents described economic as well as social influences on the adoption decision. As mentioned above, there is not only an ongoing debate about TAVI in journals but in public media as well. In particular, the public debate was perceived as generally useful, although misguided. While one respondent described the discourse as “one-sided,” focusing either on cost issues of TAVI or on its medical outcomes. The statement below indicates yet another perception:

“I’ve perceived the discussion as quite controversial and as it should be. So I thought that this was really impressive, the way that it was discussed within the society… What issues have been addressed and what has been discussed. I haven’t experienced this with other procedures….. Probably because it was done too often.” (Cardiologist 3)

The approval system for medical devices in Europe and the reimbursement system in Germany, often described as “innovation friendly” in comparison to the US medical device regulation, was a theme often brought up during the interviews. Being able to receive reimbursement at an early stage was regarded as one of the main drivers facilitating the diffusion of TAVI. The DRG rate was perceived as high, but most of the participants did not see TAVI as a way to generate as much profit as argued in the public debate. Some interviewees did say, however, that it could be a way to generate money if done in large amounts and, therefore, could be interesting for hospitals. The missing quantitative limitation regarding the maximum and minimum amount of procedures and the fact that no mandatory requirements concerning the diagnosis and infrastructure of hospitals using TAVI exist were also mentioned as factors positively influencing the diffusion rate.

## Discussion

The aim of this study was to explain the vast uptake of TAVI within the German healthcare system. Based on problem-centered interviews, it did this by applying the “innovation in health service organizations” model developed by Greenhalgh et al. to an innovative cardiac intervention in hospitals. The model was found useful to guide the research process and to structure the analysis. While the public debate centers on reimbursement policies and cost issues as main drivers for the diffusion, this study showed that multiple factors affect the spread of the procedure. Besides reimbursement policies and cost issues, the most prominent themes were the collaboration between cardiologists and cardiac surgeons, requirements needed to conduct the procedure, and the medical advantages of the method.

The collaboration between cardiologists and cardiac surgeons was identified as a factor negatively affecting the implementation and diffusion speed. It seemed that this “frozen conflict” with respect to shifting competences has reached a new level with TAVI as several respondents perceived. In some cases, the implementation of TAVI resulted in a better collaboration between both departments. The interviews indicate that this depended on central actors, which underscores previous research [22]. Champions, eager to implement the method, facilitated the dialogue between both departments and took initiative to drive the process forward. However, if the balance of power favored the standard treatment instead of TAVI, it needed stimuli like a staff turnover to facilitate new approaches. These findings suggest that moderation could be needed to foster the implementation process and to mediate between different interests. In all our cases, the procedure was implemented sooner or later, implying that if conflicts of interest existed, they were solved. The interviews suggest that cardiologists had more bargaining power. A reason for this could be the fact that the intervention is indicated by the cardiologists, and patients undergo surgery only if the cardiologist sees that as an option. Another reason lies in the possibility to hire expertise from the outside, as was mentioned in one of the interviews. Even though prevailing issues have been solved in all cases, it could lead to further dissent in the future due to expanding indications, as comments regarding this conflict imply.

According to the recommendations of German and international cardiac and cardiothoracic societies and associations, the decision whether TAVI is an option for the patient should be made by a heart team comprised of cardiologists, a cardiac surgeon, and anesthesiologists. If required, this also includes other specialists such as vascular surgeons or angiologists [27]. As the interviews indicate, this is not a given in all hospitals (see also Table 1). This refers to what Greenhalgh et al. entitle as “assimilation.” Innovations consist of a “hard core,” the innovation itself, and a “soft periphery” [20], which include organizational structures necessary to implement the innovation. Previous results have shown that the more adaptable the “soft periphery” is, the more likely an innovation will be adopted [20]. If the soft periphery, in the case of TAVI having a surgical department on site^3^, is no longer perceived as necessary, this could further increase the diffusion of the procedure. As the respondents mentioned during the interviews and as current debates suggest, this requirement is more and more questioned.

With respect to the attribute of relative advantage, which is described as “the degree to which an innovation is perceived as being better than the idea it supersedes” [19], medical advantages proved to be a strong driver. We could show that the procedure was perceived as significantly better. Still, this is only true for the current indication: older patients with severe symptomatic AS who are not suited for conventional procedures [10]. Considering the age of patients undergoing TAVI, the recommendations of the national and European cardiac and cardiothoracic societies and associations suggest a minimum age of 75 [10]. In the group of patients under the age of 75, the surgical intervention was considered superior to TAVI, although the respondents predicted changing recommendations for the near future. Experimenting with an innovation on a limited basis was labelled “trialability” by Rogers [19]. Even though experimenting with new techniques and procedures bears a high degree of risk, trialability can have an important impact on the adoption decision of medical devices and facilitate the uptake [28]. While a patient’s attitudes and beliefs can have an influence on the adoption decision and the implementation, patients either actively request new treatment methods or distrust procedures and reject them [29]. While the patient’s perception of TAVI has to be considered as an innovation-level factor, it can affect the physician’s adoption decision [16]. As pointed out by Chaudoir et al., “the patient-level factor encompasses patient characteristics such as health-relevant beliefs, motivation, and personality traits that can impact implementation outcomes.” [16].

### Recommendations for research and practice

This study raises additional questions that have to be investigated further. The ongoing debate about the indications for TAVI, as well as the requirements necessary to perform the procedure, requires more research from different angles. For instance, a discourse analysis could provide additional insights about the balance of power. This is also interesting with respect to the conflict between cardiologists and cardiac surgeons. Further studies should focus particularly on the question of how, and in which ways, new medical devices, procedures, and their diffusion affects the collaboration and interaction of medical professionalism and work processes. From a practical point of view, different factors that were discussed within this study provide opportunities to influence the implementation and diffusion of the procedure. Moderation could be a way to support the implementation process. Focus should also be placed on conflicts about competences and responsibilities as they could lead to negative implementation outcomes and, as a worst case, cause harm to the patients.

### Limitations

This study focuses only on cardiologists and thoracic surgeons working in hospitals. Further interviews with administrative staff, like medical or commercial directors or nursing staff, could provide further insights into the implementation processes. In addition, the results are mainly from the cardiologists’ point of view, and hospitals refusing to implement TAVI respectively non-adopters or “negative cases” were not included. This derived from the fact that we faced multiple challenges during the recruitment. Firstly, the subject of our research is considered controversial at the national and international level, which seemed to affect the willingness to participate in a negative way. Secondly, an analysis of the hospital’s quality reports shows the implementation of the method was consistent during the analysis period, making it hard to identify “negative cases.” Thirdly, the method of problem-centered interviews, in particular the narrative beginning, was rather unknown to the respondents. This could have caused irritation and lead some to reject participation. The respondents were also asked to recall events and discussions form the past, in some cases covering several years, which could have a negative effect on the level of detail. Even though anonymity was ensured prior to and after the interview, the answers given bear the risk of social desirability. The respondents may have given answers that they felt were pleasing to the interviewer.

## Conclusions

This paper has, based on problem-centered interviews, illustrated factors affecting the adoption, implementation, and diffusion of TAVI within German hospitals. According to the multi-level model described by Greenhalgh et al., many interacting factors influence the adoption decision, the implementation, and the diffusion of TAVI in Germany. Summarizing the results, we found that, on the innovation level, relative advantage was not only expressed in terms of medical advantages and social prestige but reimbursement also positively affected the adoption decision. The same is true with respect to the attribute of complexity: Even though the intervention was described as complex, this seems to have a positive rather than a negative effect on the adoption decision. According to the domain of communication and influence, opinion leader could be identified on the system level. The implementation of the procedure was driven by champions, typically head physicians of the department of cardiology, while cardiac surgeons took the role of gatekeepers. The readiness for innovation was reflected on the system and organizational level and referred to the conflict/cooperation between cardiologists and cardiac surgeons. A well-established cooperation supported the implementation process, whereas an internal conflict interrupted it. We could identify two system antecedents for innovation that influenced the success of the implementation: organizational self-image and the size/type of the hospital. Reimbursement policies and the public debate affected the diffusion of the method as external factors. The most dominant themes proved to be the medical advantages of the procedure, the fact that it was quite easy to have TAVI reimbursed, and the cooperation and competition between medical departments and disciplines. Interestingly, TAVI diffused relatively quickly despite major challenges and hindrances that occurred during the diffusion process. Most notably, this includes the conflict between cardiologists and cardiac surgeons regarding responsibilities and competences and, as a consequence, due to the controversial debate. Taking into consideration the professional discourse about TAVI, further research from different disciplines is needed not only to guarantee a safe and controlled diffusion of TAVI but also other emerging procedures and innovations.

## Abbreviations

AS: aortic stenosis
AVR: aortic valve replacement
DOI: diffusion of innovations
DRG: diagnosis-related group
EU: European Union
HTA: health technology assessment
TAVI: transcatheter aortic valve implantation
TAVR: transcatheter aortic valve replacement

## Footnotes

1 Also called transcatheter aortic valve replacement (TAVR).

2 From 2005 on, each acute hospital in Germany is legally obligated to publish a structured quality report on a regular basis containing information about operations and procedures that were performed. Only hospitals with more than five cases are included.

3 In this context, it is important to distinguish between an “on-site surgery” which guarantees surgical backup and an “on-site surgical department” going beyond that aspect [34].
